# Contemporary Approaches Towards the Optimization of Embryo Implantation

**DOI:** 10.3390/jcm15124723

**Published:** 2026-06-17

**Authors:** Christian Unogu, Monika Grymowicz, Anna Szeliga, Roman Smolarczyk, Anna Kostrzak, Ewa Rudnicka, Anna Duszewska, Gregory Bala, Martyna Grymowicz, Blazej Meczekalski, Eli Y. Adashi

**Affiliations:** 1 Department of Gynaecological Endocrinology, Poznan University of Medical Sciences, 60-535 Poznan, Poland; 2 Department of Gynaecological Endocrinology, Medical University of Warsaw, 02-091 Warsaw, Poland; monika.grymowicz@wum.edu.pl (M.G.); ewa.rudnicka@poczta.onet.pl (E.R.); 3 Department of Morphological Sciences, Faculty of Veterinary Medicine, Warsaw University of Life Sciences, 02-787 Warsaw, Poland; 4 UCD School of Medicine, University College Dublin, Belfield, D04 V1W8 Dublin, Ireland; 5 Medical University of Lodz, 90-419 Lodz, Poland; 6 Department of Medical Science, Brown University, Providence, RI 02912, USA; eli_adashi@brown.edu

**Keywords:** embryo implantation, uterine receptivity, recurrent implantation failure (RIF), assisted reproductive technology (ART), artificial intelligence, time-lapse imaging, endometrial receptivity analysis (ERA), microfluidic models, womb-on-a-chip, personalized reproductive medicine

## Abstract

**Background/Objectives:** Embryo implantation is a highly regulated, multistep process requiring precise synchronization between a developmentally competent blastocyst and a receptive endometrium. Despite advances in reproductive medicine, implantation failure remains a major limiting factor in assisted reproductive technology (ART), particularly in cases of recurrent implantation failure (RIF). This review aims to summarize current knowledge on the molecular, cellular, and immunological mechanisms governing embryo–endometrial interaction and to evaluate contemporary strategies for optimizing implantation outcomes. **Methods:** This narrative review synthesizes the current literature on embryo implantation, including studies addressing uterine receptivity, etiological factors contributing to implantation failure, and emerging diagnostic and therapeutic approaches. The review integrates findings from molecular biology, clinical ART practices, and bioengineering-based models. Key areas include transcriptomic tools such as endometrial receptivity analysis, time-lapse imaging, artificial-intelligence-based embryo selection, and advanced in vitro models (e.g., microfluidic “womb-on-a-chip” systems and three-dimensional embryo–endometrial platforms). The literature was identified through major biomedical databases, following a structured but non-systematic approach. **Results:** Implantation success is dependent on a complex interplay of hormonal regulation, gene expression, immune modulation, and embryo quality. Disruption of uterine receptivity during the window of implantation is a critical contributor to infertility and RIF. Multiple factors—including genetic abnormalities, maternal age, lifestyle influences, immunological imbalance, uterine pathology, and chronic endometrial conditions—are implicated in implantation failure. Emerging technologies, such as AI-assisted embryo selection, transcriptomic profiling, and advanced in vitro implantation models, provide enhanced insight into implantation dynamics and offer potential for improved clinical outcomes. **Conclusions:** Advances in understanding embryo implantation and the development of innovative diagnostic and therapeutic technologies hold significant promise for improving reproductive success. However, further research, validation, and standardization are required before these approaches can be fully integrated into routine clinical practice. A more personalized and mechanism-based approach to implantation may ultimately enhance ART outcomes and reduce the burden of infertility.

## 1. Introduction

Embryo implantation is a highly dynamic and tightly regulated biological process that requires precise temporal and spatial coordination between the developing blastocyst and the receptive endometrium. Despite decades of research, many aspects of this interaction remain incompletely understood, particularly in humans, owing to ethical and technical limitations. Recent advances in molecular biology, bioengineering, and in vitro modeling systems have provided new insights into implantation mechanisms, including cell–cell communication, immune modulation, and biomechanical signaling. These developments are essential for improving clinical outcomes in assisted reproductive technologies (ART), particularly in cases of recurrent implantation failure (RIF), which remains a major challenge in reproductive medicine.

This article presents a narrative review of the current literature, aiming to synthesize and critically discuss key mechanisms underlying embryo implantation. Unlike a systematic review, this approach allows for a broader and more integrative exploration of emerging concepts, including molecular pathways, cellular interactions, and translational perspectives relevant to clinical practice.

## 2. Search Strategy and Study Selection

**Figure 1:**
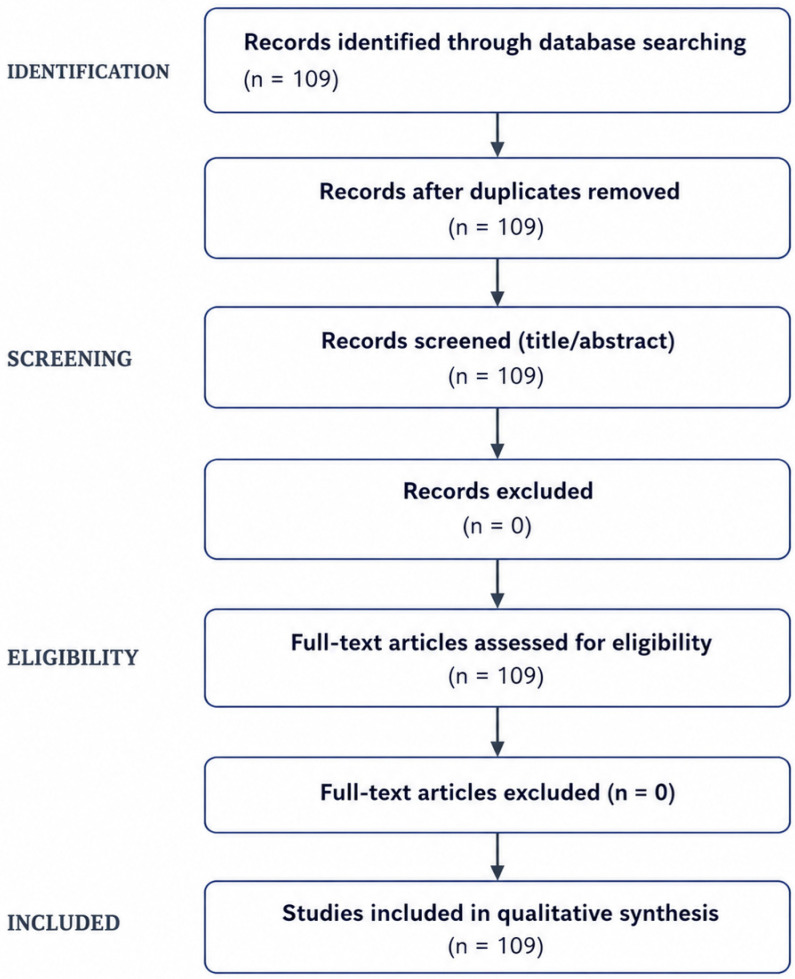
PRISMA 2020 flow diagram illustrating the study selection process for this review. The diagram outlines the literature search, screening, eligibility, and inclusion process following PRISMA guidelines.

This review was conducted in accordance with PRISMA (Preferred Reporting Items for Systematic Reviews and Meta-Analyses) guidelines [1]. A comprehensive literature search was performed using the PubMed, Scopus, and Web of Science databases up to (January, 2026) (Figure 1). Search terms included “embryo implantation”, “endometrial receptivity”, “trophoblast invasion”, “recurrent implantation failure”, and “in vitro models of implantation”.

Studies were included if they addressed human or translational implantation mechanisms, investigated molecular, cellular, or bioengineering aspects and were published in English.

Exclusion criteria included non-peer-reviewed articles and studies lacking mechanistic relevance.

PICOT Framework: P (Population): Women undergoing implantation or ART, I (Intervention): Molecular, cellular, or bioengineered models, C (Comparison): Normal vs. impaired implantation conditions, O (Outcome): Implantation success, mechanistic insights and T (Time): Not restricted.

## 3. Implantation: Basic Mechanisms and Stages

**Figure 2:**
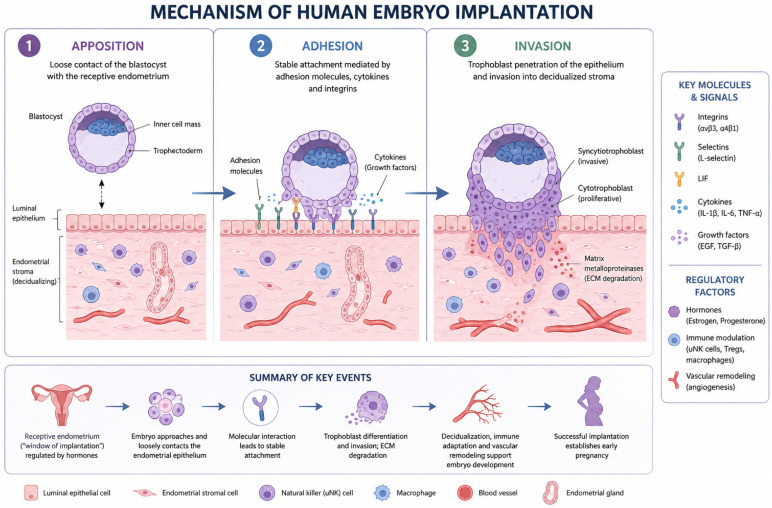
Schematic representation of the mechanism of human embryo implantation. The implantation process occurs in three sequential stages: (1) apposition, in which the blastocyst loosely aligns with the endometrial epithelium; (2) adhesion, involving firm attachment mediated by adhesion molecules, cytokines, and integrins; and (3) invasion, during which trophoblast cells penetrate the endometrial epithelium, degrade the extracellular matrix via proteolytic enzymes, and embed within the decidualized stroma. This process is tightly regulated by hormonal signaling (estrogen and progesterone), immune modulation, and embryo–endometrial cross-talk. Adapted from Norwitz et al. (2001), Dey et al. (2004), Gellersen and Brosens (2014), and Lessey and Young (2019) [2,3,5,6].

Implantation is a complex, highly coordinated process that establishes both physical and functional communication between the blastocyst and the maternal endometrium. In humans, implantation occurs approximately six to seven days after fertilization and can be divided into three sequential stages: apposition, adhesion, and invasion (Figure 2).

**Stage 1: Apposition**

During apposition, the free-floating blastocyst loosely interacts with the luminal epithelium, often at sites wherein pinopodes are present and wherein mucin expression (notably MUC1) is locally reduced. This stage is mediated by a complex interplay of cytokines, growth factors, and adhesion molecules. Leukemia inhibitory factor (LIF) and interleukin-1 (IL-1) are particularly important for initiating the embryo-endometrial crosstalk [2].

**Stage 2: Adhesion**

In the adhesion phase, integrins such as αvβ3 and α4β1, expressed on both the trophoblast and endometrial epithelial cells, stabilize embryo attachment. Osteopontin and other extracellular matrix proteins serve as bridging ligands between integrins on opposing surfaces, thereby consolidating the embryo–endometrial interface.

**Stage 3: Invasion**

The invasion phase is characterized by the differentiation of the trophoblast into cytotrophoblast and syncytiotrophoblast. For its part, the syncytiotrophoblast penetrates the luminal epithelium and the underlying stroma through regulated proteolysis involving matrix metalloproteinases (MMP-2 and MMP-9). Decidualization of the stromal fibroblasts, driven primarily by progesterone and cAMP signalling, supports controlled trophoblast invasion and modulates immune tolerance [3]. Uterine natural killer (uNK) cells and macrophages further contribute to vascular remodelling and immune adaptation, thereby ensuring successful placentation and early pregnancy maintenance [4].

## 4. Uterine Receptivity: Basic Concepts

Uterine receptivity refers to the transient state during which the endometrium is optimally prepared to accept and support blastocyst implantation. In humans, this period, known as the “window of implantation” (WOI), typically occurs between days 19 and 23 of a regular 28-day menstrual cycle and is primarily regulated by progesterone following adequate estrogen priming.

The principal role of progesterone is to induce morphological and molecular changes in the endometrium, including stromal decidualization, modulation of epithelial polarity, and altered expression of adhesion molecules and cytokines [6]. Morphologically, the receptive endometrium exhibits pinopodes, stromal oedema, and increased glandular secretion.

**Figure 3:**
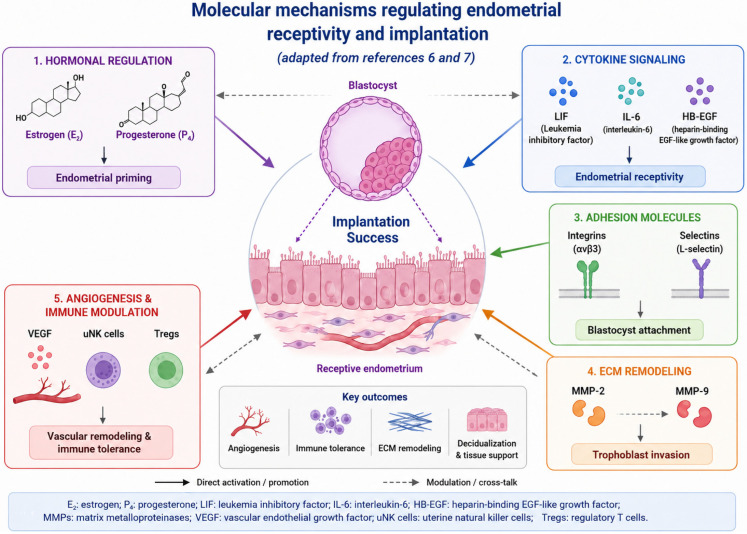
Molecular mechanisms regulating endometrial receptivity and implantation. This schematic illustrates the coordinated molecular mechanisms regulating implantation. Estrogen and progesterone prime the endometrium and regulate the expression of key cytokines, such as leukemia inhibitory factor (LIF) and interleukin-6 (IL-6), thereby enhancing endometrial receptivity and facilitating blastocyst attachment. Adhesion molecules, including integrins (αvβ3), mediate stable embryo attachment, while matrix metalloproteinases (MMPs) promote extracellular matrix remodeling to allow trophoblast invasion. Concurrently, angiogenic factors such as vascular endothelial growth factor (VEGF) support vascular remodeling, and immune cells contribute to maternal immune tolerance. Adapted from references [6,7].

At the molecular level, receptivity is characterized by the upregulation of markers such as LIF, homeobox A10 (HOXA10), integrin αvβ3, and glycodelin, along with downregulation of anti-adhesive molecules such as MUC1 at implantation sites. Transcriptional profiling has demonstrated that receptivity involves the coordinated expression of genes regulating immune modulation, angiogenesis, extracellular matrix remodelling, and cellular adhesion. Aberrant receptivity has been implicated in infertility, recurrent implantation failure (RIF), and certain cases of recurrent pregnancy loss (Figure 3). Clinical assessment methods, including histological dating, integrin evaluation, and transcriptomic tools such as the Endometrial Receptivity Array (ERA), aim to identify the optimal implantation window, although their routine clinical utility remains debated [6,7].

Proper synchronization between embryo development and endometrial maturation is critical; even a subtle temporal displacement can impair implantation success.

## 5. Implantation Failure: Causes

### Etiology and Pathophysiology

**Age and Genetic Factors**

Maternal age exerts a significant influence on pregnancy rates and on embryo quality in IVF. A study by Franasiak et al. [8] demonstrated that aneuploidy rates in blastocysts can reach 58% in women aged 40 years and 83% in women aged 43 years. Chromosomal abnormalities, including aneuploidy and structural rearrangements, are well-established contributors to early pregnancy failure, recurrent pregnancy loss, and RIF [9]. Aneuploid blastocysts demonstrate markedly reduced developmental capacity during the preimplantation stage and negligible implantation potential. Furthermore, chromosomal abnormalities including translocations, mosaicism, inversions, and deletions are more prevalent in RIF patients than in the general population [10].

**Lifestyle and Environmental Factors**

Certain lifestyle behaviours (including cigarette smoking, alcohol consumption, and obesity) have been associated with reduced ART success rates [11]. Elevated body mass index (BMI > 30 kg/m^2^) is likely associated with lower implantation rates compared with women of normal BMI (18.5–24.9 kg/m^2^) [12]. Smoking has been shown to significantly increase the risk of miscarriage; in women undergoing IVF, smoking has been associated with lower estradiol concentrations during ovarian stimulation [13]. Further evidence indicates that compounds present in tobacco smoke may interfere with corpus luteum formation and embryo implantation [14].

**Haematological Factors**

Both inherited and acquired thrombophilia have been postulated to play an important role in implantation failure [15]. The proposed mechanism linking thrombophilia to RIF involves impairment of early embryonic vascularization owing to disturbed blood flow to decidual or chorionic vessels [16]. Common inherited thrombophilias include factor V Leiden (FVL) mutation, prothrombin gene mutation, and deficiencies in natural anticoagulants such as antithrombin III and proteins C and S.

The evidence in this area remains contradictory. Qublan et al., Coulam et al., and Azem et al. reported a higher prevalence of inherited thrombophilia in women with a history of RIF [17,18,19], whereas other studies found no significant difference in prevalence compared with controls (Vaquero et al., Simur et al.) [20,21]. A recent review examining the prevalence of antiphospholipid antibodies in women with RIF similarly reported conflicting findings; however, according to ESHRE Guidelines, assessment for antiphospholipid syndrome (APS) is recommended in RIF women with additional risk factors for thrombophilia and may be considered in those without such risk factors.

**Microbiome Chronic Endometritis**

The microbiome of the female reproductive tract forms a continuum from the vagina to the fallopian tubes. Lactobacillus species constitute part of the physiologic flora of the female reproductive tract; however, infertile women frequently display abnormal vaginal microbiota, characterized by the presence of pathogenic organisms such as *Ureaplasma* spp. and Gardnerella vaginalis [22,23].

In a 2016 study, Moreno et al. demonstrated that an endometrial microbial composition rich in Lactobacillus species was associated with successful implantation, whereas endometria with a low proportion of *Lactobacillus* spp. and a high proportion of Gardnerella vaginalis and Streptococci were associated with reduced implantation rates. A subsequent meta-analysis of six cohort studies comprising a total of 1095 women reported an association between dysbiotic microbiota and impaired reproductive outcomes [24,25,26,27].

Bacterial pathogens that alter the endometrial microbiota can result in chronic endometritis (CE). Chronic endometritis, resulting from bacterial colonization of the endometrium, is frequently identified in women with a history of RIF, often in the absence of overt clinical signs of infection [28]. CE may be diagnosed by histological examination following hysteroscopic visualization or by endometrial bacterial culture [29]. In CE endometrial samples, an increased proportion of CD68+ macrophages has been observed relative to non-CE endometrium. Recent studies report a prevalence of CE of approximately 14% in patients with RIF following IVF, suggesting that CE may adversely affect endometrial receptivity [30].

**Uterine Structural Factors**

Uterine fibroids represent the most common gynaecological pathology in women, and their prevalence is higher among women with infertility [31]. Fibroids have been proposed to impair implantation through several mechanisms, including venous congestion, diminished vascular supply, increased glycodelin levels, and reduced expression of HOXA10 and IL-10 [32,33]. Endometrial polyps may similarly contribute to RIF, although further studies are needed to clarify whether polypectomy improves implantation rates [34].

Endometriosis is considered the second most common benign condition of the female genital tract after uterine myoma [35]. Pro-inflammatory cytokines, including interleukin IL-6, IL-1β, interferon (IFN)-α, and tumour necrosis factor (TNF)-α, are elevated in women with endometriosis and may disrupt the implantation process [36]. Women with endometriosis demonstrate reduced oocyte and embryo quality and quantity, lower implantation and pregnancy rates, and increased rates of spontaneous abortion [37].

**Immunological Factors**

An endometrial immune profile has been proposed to identify immune dysregulation associated with RIF [38]. Successful embryo invasion requires specific immune activation. Downregulation of Th1-type immune responses is an essential requirement for clinically normal pregnancies, while Th2-mediated responses tend to predominate during healthy gestation [39]. Compared with normal pregnancy, recurrent miscarriage and RIF are associated with significantly elevated levels of Th1 cytokines (such as interferon-gamma) and reduced serum levels of Th2 cytokines, including IL-6 and IL-10 [40].

Embryo invasion and placentation are also regulated by interactions between maternal killer immunoglobulin-like receptors (KIRs) expressed by uNK cells and fetal HLA-C molecules expressed by extravillous trophoblast (EVT). Women carrying the KIR AA genotype (two KIR A haplotypes) are at increased risk of pregnancy disorders, as the KIR AA combination generates predominantly inhibitory signals targeted at uNK cells, thereby impairing trophoblast invasion [41].

**Male factor**

By analogy with poor-quality oocytes that result in poor-quality embryos, spermatozoa of poor quality may also be responsible for low embryo quality [42]. Many factors may contribute to poor semen quality, including obesity, cigarette smoking, high caffeine intake, and alcohol consumption. These factors appear to negatively affect not only conventional semen parameters, but also molecular aspects such as sperm DNA integrity and redox status [43]. Lifestyle interventions in men can help to improve embryo quality, and it is recommended that lifestyle factors and their optimization be reviewed at the time of RIF [10].

However, although sperm DNA damage is associated with poor embryo development, conflicting data exist regarding the relationship between sperm DNA fragmentation (SDF) testing results and clinical pregnancy rates following ART [44,45]. In a recent large study by Hervas et al. comprising 1339 women, no significant difference was observed in live birth rate (LBR) per first embryo transfer or in cumulative LBR between groups with high and low SDF [46]. Similar conclusions were reached by Coughlan et al. and Bronet et al. [47,48]. Accordingly, routine testing for DNA fragmentation is not recommended by ESHRE [10].

Sperm fluorescence in situ hybridisation (FISH) is a cytogenetic diagnostic test that evaluates the frequency of chromosomal abnormalities in spermatozoa. The majority of published research has demonstrated no independent correlation between sperm aneuploidy on FISH analysis and RIF [49,50]. Consequently, sperm FISH analysis is also not recommended by ESHRE for diagnostic purposes in investigation of recurrent implantation failure [10].

## 6. Contemporary Therapies and Interventions Used in Recurrent Implantation Failure

Current attempts to manage RIF include interventions and therapies applied both prior to and during the following ART cycle. Some attempts, such as endometrial injury or using LMWH (low molecular weight heparin) in absence of thrombophilia, do not show significant improvement in ART success and thus are not recommended.

Blastocyst-stage embryo transplant compared to cleavage-state ET increases probability of implantation and is one of the possible options to be considered in RIF management. Other potentially beneficial methods include assisted hatching or preimplantation genetic testing for aneuploidies.

Based on meta-analysis from 16 studies, intrauterine autologous peripheral blood mononuclear cells (PBMC) and platelet-rich plasma (PRP) infusion are the most effective among immunomodulatory therapies and consequently are the most promising measures [51]. Certain other immunomodulatory therapies, including granulocyte colony-stimulating factor (G-CSF) administration, intravenous intralipid or IgG infusion, report notable increases in clinical pregnancy rate and live birth rate; nevertheless, they can entail serious side effects (e.g., allergic reactions, thrombosis, fluid overload).

However, due to conflicting efficacy, potential side effects and insufficient evidence, most practices still cannot be recommended within the guidelines [10]. There is still a significant need for more research to be conducted to evaluate existing treatment approaches.

## 7. Novel Tools to Improve Implantation Outcomes

### 7.1. Time-Lapse Imaging for Embryo Development Monitoring

Time-lapse imaging (TLI) is a technology applied across numerous fields and has been adopted in assisted reproduction (AR) primarily for monitoring embryos during early stages of development, though it can also be used to observe and assess sperm and oocytes [52,53,54,55,56].

Various systems exist for monitoring embryo development with respect to morphology and morphokinetics, each requiring a digital inverted microscope connected to a camera that captures images or videos at defined intervals. These systems may be integrated with an incubator, thereby maintaining embryos within a controlled environment without removal (e.g., EmbryoScope, EmbryoScope+, Geri/Geri+, Miri) or used independently (e.g., Primo Vision VO+, Eeva). Integrated systems maintain constant temperature, pH, gas composition (CO_2_ and N_2_), and in some cases humidity, creating near-ideal developmental conditions. Lighting is also an important parameter: bright-field technology (Primo Vision VO+, EmbryoScope, EmbryoScope+, Geri/Geri+, Miri) permits assessment of both morphology and morphokinetics, whereas dark-field technology (Eeva) focuses on kinetic parameters alone and provides insufficient information about embryo morphology, thereby limiting its clinical utility. In certain systems, embryos may be cultured individually to allow for personalised developmental environments [52,55,56].

Morphological and morphokinetic monitoring tracks embryo development from the zygote stage through to the blastocyst at defined time intervals. Assessed parameters include the presence of a second polar body and pronuclei, timing of cleavage, blastomere number, embryo morphology at various developmental stages, morula compaction, initiation of blastulation, and blastocyst morphology (inner cell mass, trophectoderm, and blastocoel cavity). Developmental abnormalities, including fragmentation, degeneration, vacuolization, lysis, and irregular zona pellucida, may also be identified. A comprehensive catalogue of morphological and morphokinetic parameters is provided in dedicated publications [55,56].

These parameters are monitored and analysed by TLI software in two-dimensional (2D) formats, either as time-lapse sequences or as static images. Data analysis may be performed manually (CCM-iBIS, Primo Vision VO+), semi-automatically (Miri), in a mixed manner (EmbryoScope, EmbryoScope+, Geri/Geri+, Miri), or in a fully automated fashion (Eeva) [52,55,56].

It should be noted that TLI is inherently a 2D imaging technique and does not capture the three-dimensional (3D) structure of the embryo, a limitation that is particularly relevant at the blastocyst stage. Consequently, research is ongoing into artificial intelligence (AI) algorithms capable of reconstructing 3D embryo morphology from 2D time-lapse images [57].

In addition to morphological and morphokinetic monitoring, TLI systems can support prediction of an embryo’s developmental competence [51,54,55]. It should be acknowledged that embryo assessment, selection, and prediction based solely on embryologist experience are inherently subjective and imprecise; the integration of AI as a supporting analytical tool is therefore well justified. Some studies have raised concerns regarding the clinical utility of TLI and its predictive value, highlighting the need for continued evaluation [58,59].

### 7.2. Artificial Intelligence for Embryo Selection

The application of artificial intelligence (AI) to embryo assessment and selection in assisted reproduction (AR) has been the subject of extensive review [60,61,62,63,64,65,66,67,68,69]. The general pipeline for AI-based embryo analysis encompasses data collection and preprocessing, exploratory data analysis, model selection and training, performance evaluation, model tuning, and clinical deployment.

Input data for AI models typically consists of images or videos obtained via TLI or other imaging techniques and may be supplemented with additional clinical information such as sperm and oocyte quality parameters, or implantation-associated variables with prognostic value [55,56,60]. These data are analysed using either simplified machine learning (ML) approaches or more advanced deep learning (DL) architectures, the latter now being preferred. Numerous DL models have been developed for embryo assessment and selection, and several have been integrated into regularly updated clinical applications [60,70,71].

Among a range of available models, selected examples illustrate the breadth of the field. For day 3 embryo evaluation, the Xception architecture has achieved accuracy rates of up to 98% [70]. For blastocyst grading, multiple architectures were benchmarked (including Xception, Inception V3, ResNet-50, Inception-ResNet V2, NASNetLarge, ResNeXt-101, and ResNeXt-50) with Xception demonstrating the best discriminatory performance based on morphological quality [72]. KIDScore™ (Vitrolife, Sweden) and iDAScore^®^ (Vitrolife, Sweden) were proposed as decision-support tools for blastocyst selection prior to transfer [73,74]. The MAIA platform represents another notable embryo selection application [75].

A growing subset of models has been developed not only to assess and select embryos, but also to predict biochemical pregnancy, clinical pregnancy, and live birth outcomes. These include iDAScore^®^, KIDScore™ D5, the NEQsi score, and DeepEmbryo [76,77,78,79]. Notably, AI tools have demonstrated greater accuracy than embryologists in selecting embryos for transfer [68,76,80]. AI-based classification tools for oocytes and sperm have also been developed for use in IVF [81,82], as have models supporting preimplantation genetic testing (PGT) for both monogenic and polygenic conditions [60,83]. It should be emphasised, however, that the concept of polygenic disease risk scoring remains highly controversial and currently lacks sufficient evidence to support its routine clinical application. A range of additional AI applications supports IVF across endocrinological and other clinical domains [60,67].

Nevertheless, concerns remain regarding the reliability and reproducibility of certain models, as well as their ambiguous predictive value in some contexts [69,73], potential risks in the absence of demonstrated clear clinical benefit [84], and broader ethical considerations [85]. Despite these limitations, the accuracy of current AI models for embryo assessment and selection exceeds that of experienced embryologists [68]. Although current AI tools do not yet fully meet expectations across all aspects of the IVF workflow, continued development of robust and validated models remains an important goal. At present, AI serves best as a decision-support tool to assist embryologists, clinicians, and patients in making informed, collaborative choices.

### 7.3. Gene Profiling: Endometrial Receptivity Analysis (ERA)

Endometrial receptivity is a temporally restricted state in which the endometrium permits embryo adhesion and invasion during the window of implantation (WOI). In natural cycles, the WOI typically occurs six to ten days after the luteinizing hormone (LH) surge; in hormone replacement therapy (HRT) cycles, it corresponds to five full days of progesterone exposure [86,87]. At the molecular level, this state is characterized by coordinated changes in the expression of genes involved in cell adhesion, immune modulation, cytokine signalling, steroid hormone response, extracellular matrix remodelling, and angiogenesis. High-throughput transcriptomic profiling has enabled the identification of gene expression signatures that discriminate between pre-receptive, receptive, and post-receptive endometrial phases, providing the basis for personalized embryo transfer strategies [6,88,89].

The Endometrial Receptivity Analysis (ERA) is a commercially available diagnostic test that evaluates the expression of a defined gene panel associated with endometrial receptivity using a mid-luteal endometrial biopsy. The original ERA platform was developed through microarray analysis of 238 genes differentially expressed between receptive and non-receptive endometrium, followed by computational modelling to classify endometrial samples [6]. The test is performed at the point in the cycle when the uterine lining is expected to be receptive to embryo implantation. In an HRT cycle this is 5 days after progesterone is commenced (progesterone+5) or day 7 after the LH surge is detected (LH+7) in natural cycles. The difference in timing reflects the fact that the LH surge precedes ovulation by approximately 2 days, whereas progesterone is introduced around the time ovulation would occur. If the result is non-receptive, this indicates that the patient’s WOI is displaced, either earlier (prereceptive) or later (postreceptive) than expected. In such a case, a personalized embryo transfer (pET) can be scheduled accordingly [89].

Clinical use of ERA has been evaluated predominantly in women with recurrent implantation failure (RIF). Early prospective investigations demonstrated that a substantial proportion of RIF patients exhibit a displaced WOI. Reported rates of WOI displacement vary across studies, generally ranging from approximately 20% to 30% in RIF populations; in the original cohort reported by Ruiz-Alonso et al. [89], approximately 25.9% of RIF patients were classified as non-receptive at the expected biopsy timing, consistent with WOI displacement. Importantly, when embryo transfer was adjusted to the individualized receptive window identified by ERA, implantation and pregnancy rates in previously non-receptive patients were comparable to those observed in women with standard-timing receptivity. These findings supported the biological plausibility that altered endometrial timing may contribute to implantation failure in a subset of patients. Subsequent clinical studies and observational analyses have reported similar rates of WOI displacement in RIF populations and suggested that molecularly guided timing adjustment may improve reproductive outcomes in selected cases [89,90].

More recent randomized controlled trials have questioned the routine use of ERA in unselected IVF populations. In the multicentre randomized controlled trial by Simón et al., live birth rate at the first embryo transfer did not differ significantly between ERA-guided pET and standard-timing transfer on intention-to-treat analysis; a benefit was observed only in cumulative and per-protocol outcomes over 12 months, an analytical approach that limits the strength of this conclusion [91]. A subsequent large retrospective analysis reported lower cumulative and per-transfer live birth rates when ERA was used to guide pET after a previously failed transfer [92]. These findings have prompted ongoing debate regarding the cost-effectiveness and clinical utility of ERA outside of defined patient subgroups. Systematic reviews and meta-analyses conclude that, while ERA may identify a displaced WOI in a minority of patients, robust evidence to support its universal application is lacking, and its routine use outside defined subgroups (notably RIF) remains debated [91,92].

From a methodological perspective, transcriptomic endometrial profiling represents a significant advance over histological dating, which has demonstrated limited reproducibility and poor correlation with fertility outcomes [93]. Molecular dating approaches, including ERA and other RNA sequencing-based platforms, offer objective and reproducible classification grounded in gene expression patterns rather than subjective morphological criteria. Nevertheless, several sources of variability must be considered when interpreting ERA results, including the route of progesterone administration, the precision of biopsy timing, and embryo quality. Of particular note, the reproducibility of receptivity classification is imperfect: discordant results have been reported between repeated cycles and, in some studies, across laboratories and transcriptomic platforms, which constrains the reliability of ERA as a fixed determinant of the WOI [93].

In summary, ERA represents an important integration of transcriptomics into reproductive medicine, offering a personalized approach to embryo transfer timing by molecularly characterizing the WOI. While biologically plausible and potentially beneficial in selected patients (particularly those with RIF), its routine use in all IVF patients remains controversial. Further high-quality randomized trials and standardized definitions of implantation failure are required to clarify the optimal clinical indications and long-term reproductive outcomes associated with gene expression-based endometrial receptivity testing.

### 7.4. Microfluidic “Womb-on-a-Chip” Models

Microfluidic womb-on-a-chip systems represent an innovative class of organ-on-a-chip technologies that replicate key features of the human uterine microenvironment with high physiological fidelity in vitro, enabling the study of embryo implantation and its failure. These platforms integrate microfluidic channels, three-dimensional (3D) architecture, and multicellular constructs, thereby facilitating controlled fluid flow, biochemical gradients, and tissue-level interactions that are difficult to achieve in traditional static culture systems. This enhanced microenvironment allows researchers to capture dynamic cellular behaviours critical to implantation success and to model reproductive disorders such as RIF with considerable precision [94,95,96].

A recent high-impact development in this field is a 3D in-chip implantation model constructed by combining bioengineered human endometrial tissue with human blastocysts or stem cell-derived blastoids on a microfluidic chip. This system successfully recapitulates the principal stages of human embryo implantation (apposition, attachment, and invasion) followed by early post-implantation development, overcoming major limitations of 2D culture systems and ethical constraints associated with in vivo studies [94,96]. Importantly, chips constructed using cells from patients with RIF exhibited reduced blastoid implantation capacity compared with models derived from fertile individuals, demonstrating the platform’s ability to faithfully mirror clinical infertility phenotypes [94,96].

Beyond mechanistic investigation, these models are proving valuable for therapeutic discovery. In one 3D microfluidic system, screening of over 1000 FDA-approved drugs identified compounds that improved implantation outcomes, thereby highlighting the potential of womb-on-a-chip technologies to inform personalised treatment strategies for patients with implantation disorders [94,96]. This approach represents a shift from empirical ART practices towards evidence-based, patient-specific optimization.

Further advances include the creation of endometrial epithelial monolayers on microfluidic chips using patient-derived organoids. These engineered layers maintain physiological histological features, hormonal responsiveness, and barrier integrity, providing platforms for longitudinal study of endometrial receptivity and host–embryo interactions [95]. Such models enable investigation of how stromal and immune components contribute to implantation success and may help identify molecular drivers of receptivity or resistance to trophoblast invasion.

Enhanced microfluidic platforms can also evaluate endometrial receptivity and trophoblast invasion processes in detail, enabling analysis of the maternal–foetal interface and identification of signalling pathways critical for implantation [96]. These engineered systems thus provide not only mechanistic insight but also a dynamic platform for assessing candidate interventions (such as growth factors, cytokines, or small molecules) that may improve the likelihood of successful embryo attachment and pregnancy.

**Figure 4:**
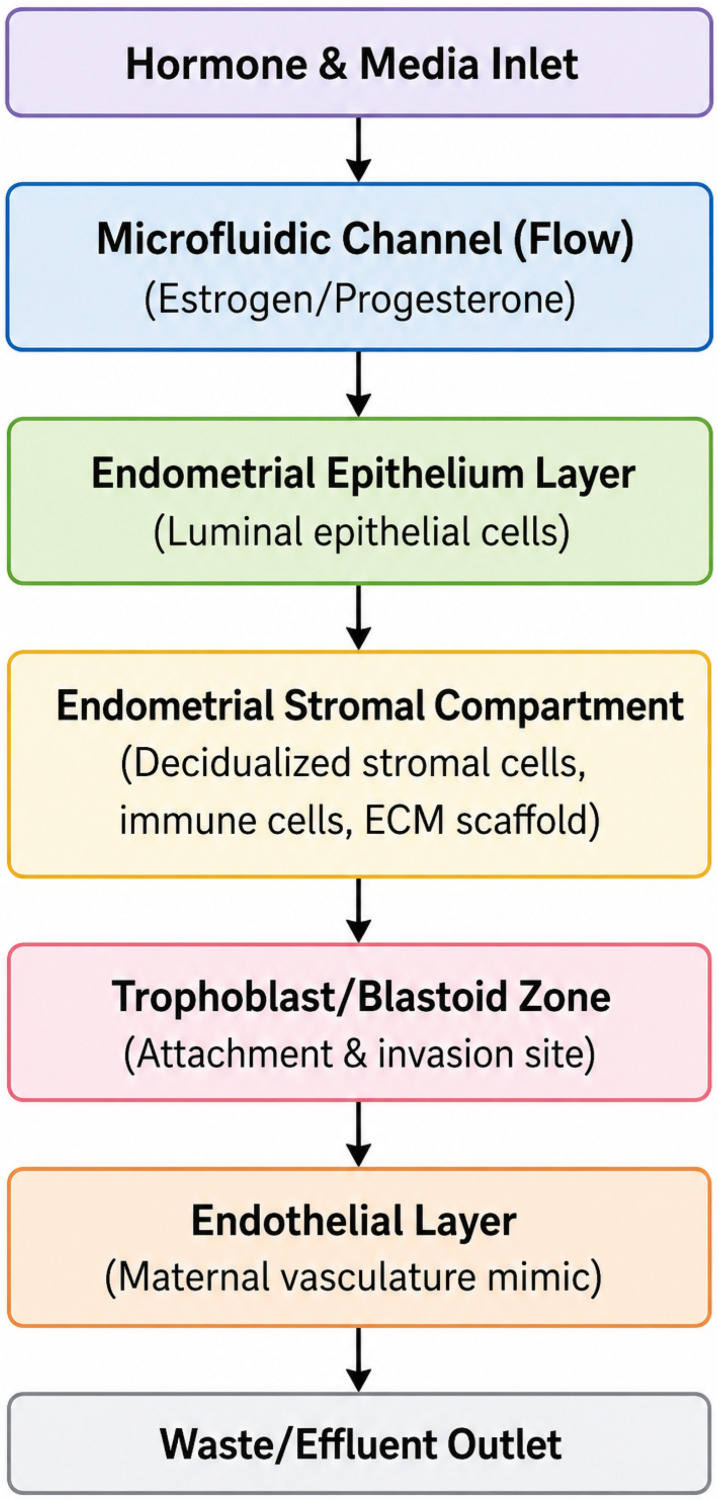
Schematic representation of a microfluidic “Womb-on-a-Chip” model, illustrating the layered compartmental architecture from hormone and media inlet through to the waste/effluent outlet. Sequential layers include the microfluidic channel (delivering estrogen and progesterone), the endometrial epithelium layer (luminal epithelial cells), the endometrial stromal compartment (decidualized stromal cells, immune cells, and extracellular matrix scaffold), the trophoblast/blastoid zone (site of attachment and invasion), and an endothelial layer representing the maternal vasculature (adapted from Li et al., Cell, 2026 Ref. [92]).

Recent advances in microfluidic womb-on-a-chip platforms have enabled the reconstruction of the human implantation microenvironment, integrating epithelial, stromal, trophoblast, and vascular compartments under dynamic hormonal flow (Figure 4). These systems closely recapitulate the stages of implantation and provide powerful tools for investigating RIF and for developing personalised therapeutic strategies [94,97].

Microfluidic womb-on-a-chip models are reshaping our understanding of human embryo implantation by offering physiologically relevant, ethically permissible, and highly controllable experimental platforms. They bridge the gap between in vitro culture and in vivo complexity, providing powerful tools to investigate implantation failure, screen therapeutics, and advance personalised reproductive medicine.

### 7.5. Microengineering: Real-Time Recording of Human Embryo Implantation

Failure of uterine implantation is one of the principal causes of infertility, accounting for approximately 60% of spontaneous abortions. Until recently, it had not been possible to observe this process in humans in real time. What limited information was available had been derived from static images captured at discrete time points.

A landmark advance in the study of embryo implantation was achieved in August 2025, when scientists from the Institute of Bioengineering of Catalonia (IBEC) and Dexeus University Hospital reported the first successful real-time, three-dimensional recording of the human implantation process [98]. Using a novel ex vivo implantation platform compatible with high-resolution microscopy, the researchers employed traction force microscopy of live mammalian embryos (both mouse and human) to investigate the role of mechanical forces and mechanosensitivity in the implantation process. Their platform enabled continuous tracking of embryonic development for up to six days following implantation, which in humans typically occurs approximately five days after fertilization.

This experiment revealed a key milestone in early human development: the precise moment at which the newly formed embryo attaches to the uterine lining [98]. The findings significantly advance our understanding of the mechanisms governing embryo implantation and have important implications for improving fertility rates and optimizing ART procedures.

Notably, it was demonstrated that human embryos exert traction forces on their surrounding environment, inducing tissue remodelling at the implantation site. Comparative analysis of human and mouse embryo implantation revealed a fundamental species-specific difference: whereas the mouse embryo adheres to the surface of the uterine epithelium, the human embryo penetrates the uterine tissue entirely before proliferating from within. Additionally, the human embryo secretes enzymes that degrade surrounding tissue during the invasion process.

Of particular clinical relevance was the comparison of implantation dynamics in embryos from younger and older mothers. Embryos from older mothers displayed increased contractility in their outer trophoblast tissue, which was associated with defective implantation. This finding identifies a potentially novel pathway for improving ART success rates in older women. Future research by Godeau and colleagues aims to elucidate how additional parameters (including extracellular matrix stiffness and embryo invasion depth) influence the mechanics of implantation.

## 8. Novel In Vitro Models of Implantation

Faithful in vitro models are essential for advancing our understanding of implantation and early post-implantation embryo development. In vitro models currently used for implantation research are presented in the Table 1.

### 8.1. Embryo Models

Access to human embryos for experimental purposes is severely constrained by ethical and legal frameworks. Although animal embryos are more readily available, significant interspecies discrepancies in implantation strategies among mammalian species limit their translational utility.

The development of blastoids (blastocyst-like, self-organized structures derived from human pluripotent stem cells) has created new opportunities to investigate implantation and early pregnancy processes, with the potential to address fundamental research questions prior to testing on true embryos [99]. Blastoids are not yet a perfect replica of native embryos; they lack the earlier developmental stages from fertilization through cleavage, during which genomic imprinting and critical cell fate decisions occur. Nevertheless, these organoids closely resemble preimplantation blastocysts and can be induced to develop structures analogous to the gastrulating embryo, thereby forming both embryonic and extra-embryonic germ layers.

### 8.2. Endometrial Models

The endometrium comprises a luminal layer of polarized epithelial cells supported by an underlying stromal–vascular compartment, undergoing cyclical changes orchestrated by ovarian steroid hormones. Endometrial cells may be obtained by endometrial biopsy or from cells isolated from menstrual effluent.

Two-dimensional (2D) models have demonstrated that interaction with endometrial stromal cell monolayers prevents apoptotic activity in blastoid cells [100], providing early mechanistic insights into embryo–endometrial cross-talk. More recently, a substantial transition from 2D to three-dimensional (3D) biological models has been observed (Table 1). Early endometrial organoids (EOs) were limited by the inward-facing orientation of the apical surface within the organoid lumen. Advances in culture methodology have enabled the generation of polarity-reversed endometrial epithelial organoids that more closely reflect native tissue architecture [101,102]. These models have confirmed the role of endometrial stromal cells in promoting trophoblast growth and invasion and have demonstrated that the luminal epithelium directs the orientation of blastoids during implantation [103].

Optimized matrix support and culture media conditions are required for the continued development of 3D in vitro endometrial assembloids that more comprehensively represent the tissue complexity of the superficial endometrial layers. The extracellular matrix (ECM), composed of protein fibres and fibrils within a hydrated glycosaminoglycan network and containing numerous cytokines, growth factors, and regulatory proteins, exerts significant influence over cellular behaviour [104]. Biomaterials that mimic the endometrial ECM may be of animal origin (collagen, gelatin, Matrigel), plant origin (alginate, agar), or synthetic composition (polyethylene glycol). Hybrid or composite formulations combining two or more biomaterials may be employed to enhance mechanical properties and biocompatibility. Proteins extracted from decellularized endometrial tissue improve biochemical similarity to native tissue composition [105].

To enable in vitro observation of all stages of implantation and early post-implantation development of human embryos and blastoids, a cell-engineered receptive endometrial scaffold technology (CREST) was developed. This scaffold models both the epithelial and stromal compartments of the superficial layer of the receptive human endometrium [93].

Traditional static culture systems have increasingly been supplemented or replaced by microfluidic on-a-chip devices, which create microphysiological systems capable of modelling inter-organ communication. A particularly significant advance in this technology is an in vitro implantation model that combines human blastoids or blastocysts with 3D bioengineered human endometrial tissue (integrating luminal and glandular epithelial cells and stromal cells within hydrogel and Matrigel matrices) within microfluidic chip devices [106]. This platform has enabled detailed transcriptomic profiling of blastoid–endometroid interactions and the identification of several ligand–receptor pairs implicated in early implantation events. Additionally, differences in implantation dynamics between blastoids in RIF-derived and control-derived endometrioids were characterized. Future research employing this model will enable testing of candidate compounds with potential to improve implantation outcomes.

### 8.3. Role of Stem Cells and Exosomes in Embryo Implantation

Stem cells and extracellular vesicles, particularly exosomes, are emerging as key regulators of embryo–endometrial communication. Endometrial stem/progenitor cells contribute to cyclical regeneration and maintenance of endometrial receptivity and are capable of differentiating into epithelial and stromal components, thereby supporting implantation.

Exosomes derived from both trophoblasts and endometrial cells mediate intercellular communication by transferring proteins, lipids, and nucleic acids, including microRNAs. These vesicles influence critical processes such as immune modulation, angiogenesis, and extracellular matrix remodelling. For instance, trophoblast-derived exosomes may promote immune tolerance by modulating uterine natural killer (uNK) cells and macrophages, thereby facilitating successful implantation.

Recent studies also suggest therapeutic potential: where stem-cell-derived exosomes may improve endometrial receptivity in patients with recurrent implantation failure, although further research is needed to clarify their clinical applicability and underlying mechanisms. Recent studies support the role of regenerative and extracellular vesicle-based mechanisms in reproductive biology [107,108,109].

### 8.4. Future Perspectives

Development continues in search of physiologically relevant embryo–endometrial models that provide insights into human implantation and early pregnancy. Optimization and standardization of these models are essential to prevent misleading or erroneous interpretation of results. As with blastoid models, standardization will need to encompass not only specific cell lineages, but also extracellular matrix composition and culture media components that may influence differentiation and implantation responses. From an endometrial perspective, the development of more comprehensive models incorporating immune and vascular cell types to generate more physiologically representative systems remains a priority [106].

The progressive development of relevant embryo–endometrial models holds considerable promise for more refined investigation of implantation dynamics, and for the establishment of scalable platforms for drug screening as well as the development of personalised therapeutic interventions.

Based on the mechanisms and evidence discussed, key clinical recommendations to improve implantation outcomes are summarized in Table 2.

## 9. Conclusions

Implantation of the human embryo into the endometrium is the crucial event for the establishment of a healthy pregnancy. The process of implantation during which the blastocyst attaches to the decidualizing endometrium is a multi-stage and highly coordinated procedure with sequential stages of apposition, adhesion and invasion. Human reproduction is defined by a high failure rate, and despite all the conducted research, there is still limited knowledge about the earliest stages of the embryo–maternal-endometrium interaction. The multiple genetic, immunological, hormonal, structural and haematological factors affecting implantation may contribute to the occurrence of unexplained infertility, recurrent implantation failure, early pregnancy loss, intrauterine growth restriction or pregnancy induced hypertension. Therefore, new techniques enabling time-lapse imaging, application of artificial intelligence to embryo assessment and selection, and genetic profiling are currently used in the hope of improving implantation rates. Moreover, new advanced embryo–endometrial models that provide insights into crosstalk between the trophoblast and the endometrial compartments hold considerable promise for a deeper understanding of the process, and for the establishment of platforms for drug screening and for the development of personalised therapeutic interventions.

## Figures and Tables

**Table 1:** Embryo–endometrium interaction models.

Key Findings	Construction	Embryo–Endometrial Model
Endometrial stromal cell monolayers prevented apoptotic activity in blastoid cells	Blastoid/blastocyst interacting with endometrial stromal cell monolayer	2D model
Demonstrated the role of the luminal epithelium in directing blastoid orientation during implantation	Blastocyst/blastoid interacting with spherical endometrial organoid	3D organoid/organoid model
In vitro observation of all stages of implantation and early post-implantation development	Blastocyst/blastoid interacting with assembloids representing superficial endometrial layers, including epithelial and stromal cells and extracellular matrix	3D organoid/endometrial assembloid in static culture
Real-time observation of implantation dynamics Transcriptomic profiling of blastoid–endometroid interactions; identification of ligand–receptor pairs in early implantation; characterization of implantation dynamics in RIF vs. control-derived endometrial assembloids	Blastocyst/blastoid interaction with assembloids of superficial endometrial layers (epithelial and stromal cells, extracellular matrix) within microfluidic on-chip devices	3D microfluidic on-a-chip devices
Potential for more refined studies of implantation dynamics; Scalable platform for drug screening and personalized therapeutic interventions	-Standardization of blastoid formation. -Standardization of extracellular matrix and culture media components. -Incorporation of vascular and immune cell components into endometrial assembloid models.	Future perspectives

**Table 2:** Key Clinical Recommendations for Improving Embryo Implantation Outcomes.

Clinical Implication	Recommendation	Domain
Improves synchronization between embryo and endometrium	Assess timing of implantation window (e.g., personalized embryo transfer)	Endometrial Receptivity
Enhances implantation success and reduces failure rates	Prioritize high-quality blastocysts and consider genetic screening where appropriate	Embryo Quality
May improve embryo tolerance and implantation success	Evaluate and manage abnormal immune responses in selected RIF patients	Immune Modulation
Optimizes implantation conditions	Identify and treat uterine pathologies (e.g., polyps, fibroids, chronic endometritis)	Uterine Environment
Supports personalized treatment strategies	Consider emerging biomarkers of receptivity and implantation	Molecular Signalling
Potential future therapeutic option (requires further evidence)	Explore experimental use in thin endometrium or refractory implantation failure	Stem Cells and Regenerative Approaches
Promising diagnostic and therapeutic applications	Investigate role in embryo-endometrium communication (research setting)	Exosomes
Improves overall reproductive outcomes	Address BMI, smoking, stress, and metabolic conditions	Lifestyle & Systemic Factors

## Data Availability

No new data were created or analyzed in this study.
